# [Retracted] Sensitization strategies in lung cancer

**DOI:** 10.3892/ol.2025.15198

**Published:** 2025-07-21

**Authors:** Xiao-Ying Zhang, Peiying Zhang

Oncol Lett 12: 3669–3673, 2016; DOI: 10.3892/ol.2016.5146

Following the publication of this article, an interested reader drew to our attention that the flow of the text, and the wording of numerous passages of text within the article, bore striking and unexpected similarities to the wording employed in a PhD thesis by Katarzyna Zielinska-Chomej of the Karolinska Institutet, Stockholm, in 2015 called “Analysis and characterization of chemo- and radiation therapy sensitizing strategies in tumours with focus on effects of phenothiazines on DNA damage response signalling”.

After having conducted an internal investigation, the Editor of *Oncology Letters* has determined beyond all reasonable doubt that the review submitted to our Journal shared an unacceptable level of identity with the PhD thesis by Katarzyna Zielinska-Chomej; therefore, this review article is to be retracted from the Journal. The Editor of *Oncology Letters* deeply regrets that this article was not intercepted by our own checks for plagiarism prior to the publication of the review, and sincerely apologizes to the author of the PhD thesis and the readership for the inconvenience caused. We also thank the interested reader for bringing this matter to our attention.

